# Aggregate web activity dataset for user-agent behavior classification

**DOI:** 10.1016/j.dib.2025.111297

**Published:** 2025-01-16

**Authors:** Geza Lucz, Bertalan Forstner

**Affiliations:** Department of Automation and Applied Informatics, Faculty of Electrical Engineering and Informatics, Budapest University of Technology and Economics, Műegyetem rkp. 3., H-1111 Budapest, Hungary

**Keywords:** AI training, Bot detection, Exploit detection, Online resource management

## Abstract

600 million web access requests made to multiple servers have been collected between 2019 and 2023. The 4-year automated collection spans over 8000 domains and had iteratively been upgraded with extra data fields up until its closure in March of 2023. The dataset is normalized and highly expandable though the fractal tree index facilities provided by MySQL and the TokuDB storage engine. It is suitable for researching web browser user-agent information-based behavior and constructing or verifying strategies for exploit and bot identification. The large sample size makes it a good choice for AI training and provides a unique opportunity to track the long-term evolution of specific user-agents and their originating IP address ranges.

Specifications Table

**Table:** 

Subject	Computer Networks and Communications, Data Mining and Statistical Analysis, Applied Machine Learning
Specific subject area	Network Traffic Analysis (NTA)
Type of data	MySQL database dump
Data collection	The data had been collected from Apache 2.x web server logs generated by 8780 websites distributed across 10 servers. Daily service logs were collected and normalized in batches. The normalization process used the visitor actions as the base entity and its properties as the attributes. These attributes included initially: user-agent, originating IP address country, last 10 characters of the query string, response status and had been extended with anonymized data: originating IP address and target domain name. Anonymization in this context means that the data can be used to determine matches across the database, but the original value is masked.
Data source location	Hungary, but the data is not location specific
Data accessibility	Repository name: Zenodo Data identification number: 10.5281/zenodo.14497695 Direct URL to data: https://zenodo.org/records/14497695 Instructions for accessing these data: The data is compressed with bzip2. It can be uncompressed to its 490GB original with bzip2, 7-zip or WinZip
Related research article	

## 1 Value of the Data

• The database fully preserves the user-agent strings of all web hits as well as access records to the robots.txt files. RFC 9309 compliant bots adjust their behavior to follow robot.txt instructions and usually identify themselves as bots in the agent-string. Therefore, tracking the individual long-term RFC 9309 compliance behavior of self-identified bots becomes possible.
• Based on the activity patterns of self-identified bots behavior classification schemes can be constructed to further identify hidden bots, using methods that may include statistical analysis or machine learning or both.
• The database is normalized along multiple properties of web visits, including source, access methods and response code, etc. For limited and well understood subsets, this allows a single component to be analyzed via statistical or expert knowledge-based methods [1] and be used as label in a supervised learning scenario for the remaining reduced feature-vector space. The gained insight then can be leveraged for unlabeled cases in this or other third-party datasets [2].
• The database spans over 4 years of data and thus makes the long-term lifecycle analysis of specific web browser versions more readily available as compared to shorter surveys [3].
• The lifecycle analysis of web browsers allows the comparison of browser update strategies as well as their simulational modelling [4].
• Simulational modelling of browser lifecycles allows for the search of agent-strings in the database that either show high or low correlation to modelled behavior. Explaining these discrepancies is a data security and research challenge in each separate case.

Detecting bot-like behavior for non-self-reporting agents and for those that do not exhibit RFC 9309 compliant behavior allows the construction of simple firewall or rate-limit rules to augment OWASP ModSecurity that has a longer update cycle.

## 2 Background

Online service providers are contractually obligated to maintain a pre-determined SLA for their customers’ online services. Public facing services such as websites are increasingly abused by third party actors [5]. These include agents that generate traffic that is not beneficial to the service owners. Some examples are unauthorized AI training bots, SEO spiders, intelligence agents and any kinds of exploits [6]. Besides the service security implications these activities greatly increase the cost and complexity of maintaining the expected service availability levels.

While some bots adhere to RFC 9309, some do not. The primary goal of this dataset is to allow the creation of schemes, methods and toolsets to aid in the faster discovery of non-RFC 9309 compliant bots. Once identified, forcing their QoS to levels allowed in the robots.txt file would result in a simpler and more manageable SLA/Cost function.

## 3 Data Description

**Fig. 1:**
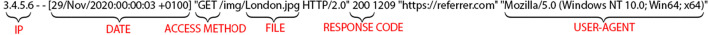
Apache web server log structure.

The database is the normalized collection of standard Apache web server logs (Fig. 1). Each line in such a log is called a hit and is generated when a visitor makes a request to any online resource on the web server. These hits contain information about the web visit including the originating IP address, the resource requested, information about the client software and the server response code

Using individual logs in their original format is tedious especially because each file contains a limited time interval or had been collected from a single domain or web server. Moreover, the agent strings are text based, allowing for only CPU intensive text processing.

**Fig. 2:**
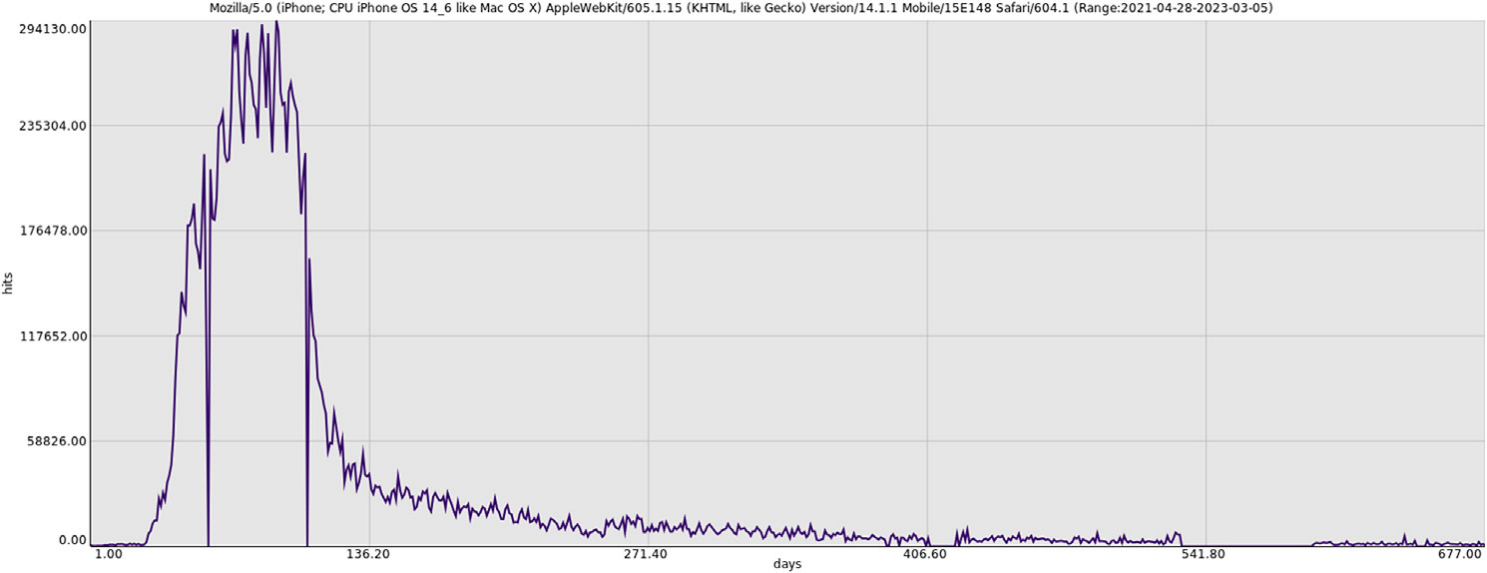
Lifecycle of a specific user-agent, reporting hit intensity versus days elapsed since first observation.

However, our aggregated and structured data allows quick access to custom filtered datasets, for example the usage intensity lifecycle of a specific user-agent as seen of Fig. 2.

**Fig. 3:**
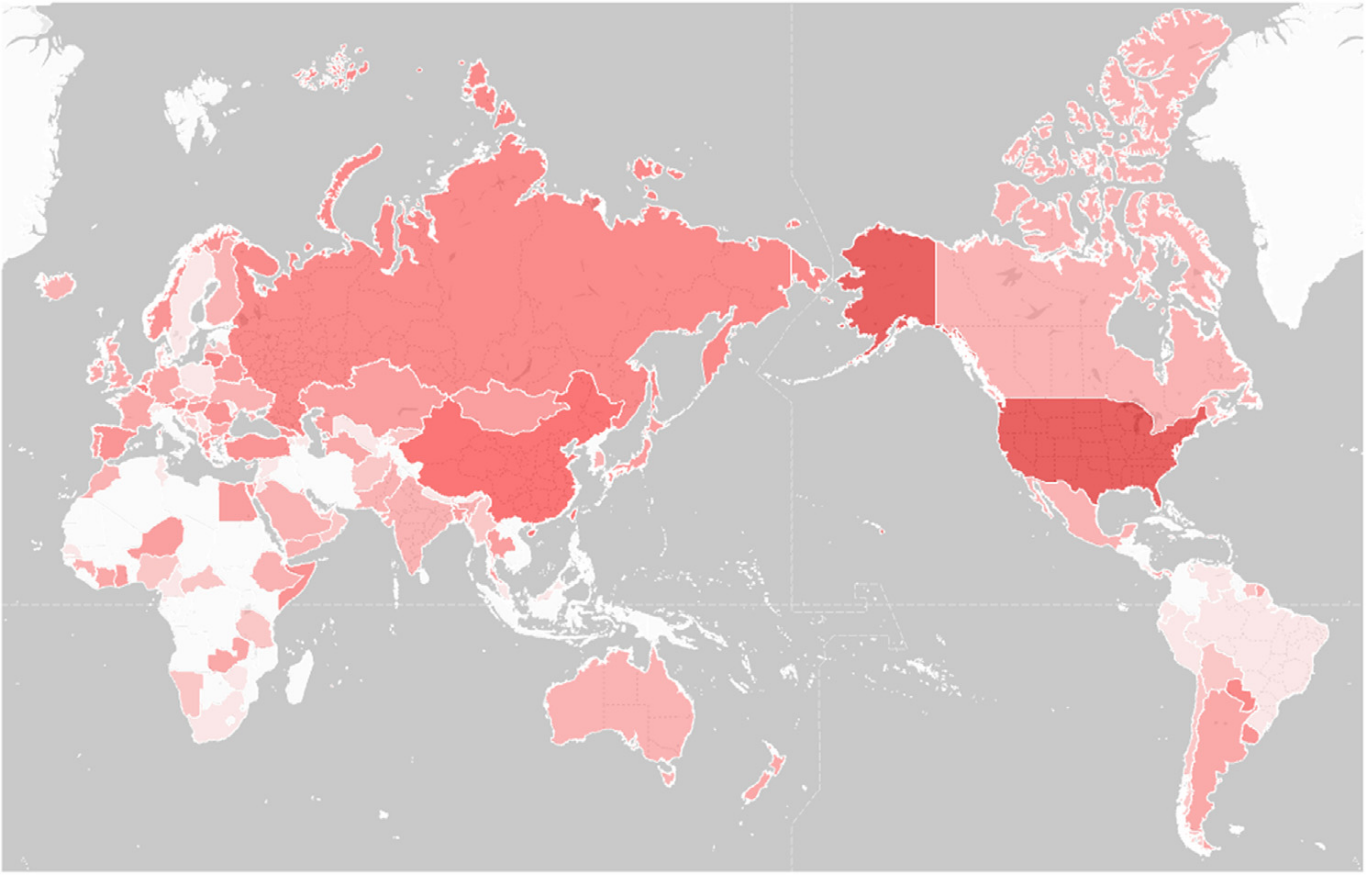
Likelihood of a specific user-agent being used in different countries. Deeper red represents an elevated likelihood.

The ability to query the source location of hits also allows the generation of originating geographical heat maps for as seen on Fig. 3. for that same user-agent between custom start and end dates.

### 3.1 Technical details

**Fig. 4:**
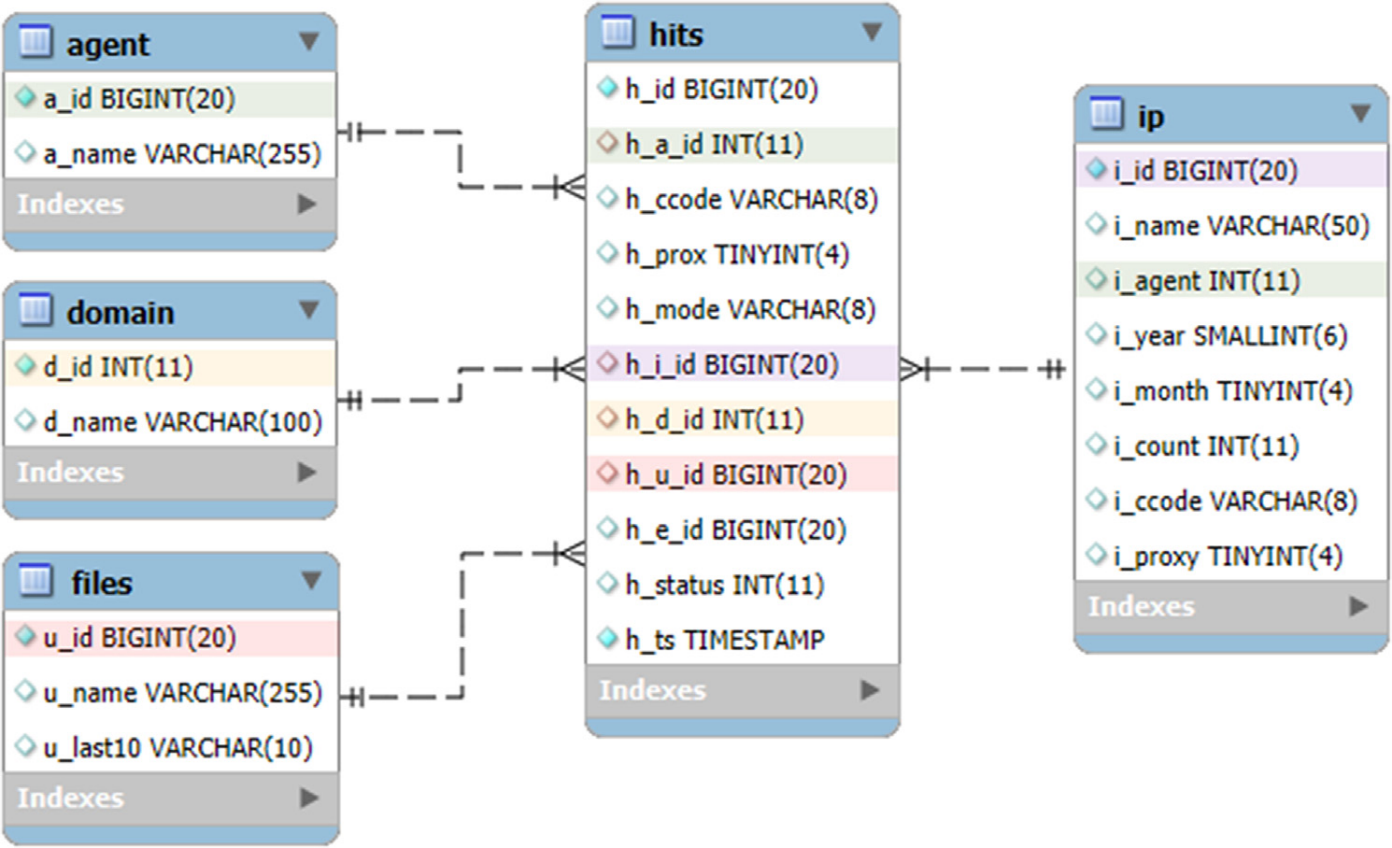
Table and field structure of the normalized user-agent database.

Our data is in a standalone MySQL database that uses the TokuDB storage engine. The full structure is shown on Fig. 4.

TokuDB is available as open source, but it is advised to use a Linux or MySQL distribution that already supports it as a prebuilt plugin (https://mariadb.com/kb/en/installing-tokudb/).

It is possible to manually alter the database dump file to use a different storage engine, but in our experience, no other storage engine supports this amount of data with our indexing scheme.

### 3.2 Hits table

The data is organized around the hits table. Each entry describes a web-based visit from a remote agent to one of the monitored domain websites.

**Table:** 

Column name	Usage / explanation
h_id	Primary key
h_a_id	Foreign key to agent table
h_ccode	ISO 3166 – 2 letter country code of originating ip on record
h_prox	NOT USED
h_mode	Request method (filled out beginning in 2021)
h_i_id	Foreign key to ip table
h_d_id	Foreign key to domain table
h_u_id	Foreign key to url table (filled out beginning in 2021)
h_e_id	NOT USED
h_status	Original RFC 7231 server response code
h_ts	Timestamp of the insertion (not the timestamp of the request)

### 3.3 Agent table

This table collects the agent strings extracted from the requests. All strings are unique. When the hit is locally initiated and the agent-name contains a domain name, those strings have been encoded with a sequence of hash functions.

**Table:** 

Column name	Usage / explanation
a_id	Primary key
a_name	User-agent string

### 3.4 Domain table

This table collects the domain name strings extracted from the requests. All name strings are unique and have been encoded with a sequence of hash functions.

**Table:** 

Column name	Usage / explanation
d_id	Primary key
d_name	Domain name string

### 3.5 Files table

This table collects the file names from the query string portion of the request. All name strings are unique and have been encoded with a sequence of hash functions. The last 10 characters are stored in cleartext to allow file type identification.

**Table:** 

Column name	Usage / explanation

u_id	Primary key
u_name	File name string
u_last10	last 10 characters of u_name in cleartext

### 3.6 Ip table

This table collects the IP addresses. For faster processing the i_name,i_agent,i_year,i_month group is handled as a compound key. This means that even if an IP address had been observed before, a new record is inserted if it came with a different agent-string or in a different year or month. The IP addresses have been encoded for GDPR compliance with a method that still allows equivalence checking. The corresponding geolocation information is in both the hits and ip tables to allow for less join operations and thus faster processing.

**Table:** 

Column name	Usage / explanation
i_id	Primary key
i_name	IP address
i_agent	Foreign key to agent table
i_year	Year of the record processing
i_month	Month of the record processing
i_count	Number of references in the hits table
i_ccode	ISO 3166 – 2 letter country code of originating ip on record
i_proxy	NOT USED

Number of records in each of the tables:

hits: 6052947475

agent: 2274125

domain: 8780

url: 71204238

ip: 52824895

## 4 Experimental Design, Materials and Methods

The data were collected directly from web servers after their daily rotation by the processing server. Usually, web servers create a separate log for each hosted domain. Once all logs had been collected, they were batch processed by the following script. This means that each log file had been processed individually by executing the proclog.pl file with the name of the log file given as an argument:

proclog.pl domain_log.202412

The proclog.pl script had been published on Github, complete with installation and quickstart instructions:

https://github.com/glucz/normalized_apache_log_collection [7]

Prior to running the script, the database and credentials must be set up and configured. The database structure is also part of the repository. The researcher has the option to expand our published database or create a new collection from the blank schema files.

## Limitations

Due to data type differences: h_a_id (hits table): int versus a_id: bigint (agent table), once the agent key exceeded 2147483647, the hits for those agents were not recorded.

Due to data type differences: i_agent (ip table): int versus a_id: bigint (agent table), once the agent key exceeded 2147483647, the ip for those agents were not recorded.

This means that only agents with a_id of 2147483647 and below have ip and hits data.

The data collection was fully automated at an offline location requiring scheduled data transfers. On some days, technical difficulties occurred, and for those days data are missing from the aggregate. On other days, scheduler conflicts occurred, and their data was processed together with the next day's batch. This is noteworthy as for GDPR considerations the actual time of the hits was replaced with the date and time of their normalization on the next day.

## Ethics Statement

Authors have read and agree to abide by the ethical requirements for publication in Data in Brief and confirm that the current work does not involve human subjects, animal testing, or data collected from social media platforms.

## CRediT Author Statement

**Géza Lucz:** Data curation, Investigation, Methodology, Software. **Bertalan Forstner:** Editing, Supervision.

## Data Availability

ZenodoWeb browser useragent and activity tracking data (Original data).

## References

[1] Alam S., Dobbie G., Koh Y.S., Riddle P. (2014). Proceedings of the 2014 IEEE Congress on Evolutionary Computation.

[2] H. Qin, X. Zhan, Y. Li, Y. Zheng, (2024). FlexSSL: a generic and efficient framework for semi-supervised learning. 10.3233/FAIA240648.

[3] M. Inoue, M. Hashimoto, (2020). A research of HTTP request and an identification method of fake User-Agent values (Vol. 119) [IEICE Tech. Rep]. https://ken.ieice.org/ken/paper/20200303m1ve/eng/.

[4] Raza A., Hussain M., Tahir H., Zeeshan M., Raja M., Jung Ki-H. (2024). Forensic analysis of web browsers lifecycle: a case study. J. Inf. Secur. Appl..

[5] Dave M., Dodiya K., Akash K., Patel D. (2024). Bot detection in motion: real-time network traffic insights. Int. J. Innovat. Res. Comput. Commun. Eng..

[6] Rahman R., Tomar D. (2021). Threats of price scraping on e-commerce websites: attack model and its detection using neural network. J. Comput. Virol. Hack. Tech..

[7] glucz (2024). glucz/normalized_apache_log_collection: Zenodo release V1.1 (V1.1z). Zenodo.

